# Prolactin-induced PAK1 tyrosyl phosphorylation promotes FAK dephosphorylation, breast cancer cell motility, invasion and metastasis

**DOI:** 10.1186/s12860-016-0109-5

**Published:** 2016-08-20

**Authors:** Alan Hammer, Maria Diakonova

**Affiliations:** Department of Biological Sciences, University of Toledo, 2801 W. Bancroft Street, Toledo, 43606-3390 OH USA

**Keywords:** PAK1, FAK, Prolactin, Tyrosyl phosphorylation, Breast cancer cells

## Abstract

**Background:**

The serine/threonine kinase PAK1 is an important regulator of cell motility. Both PAK1 and the hormone/cytokine prolactin (PRL) have been implicated in breast cancer cell motility, however, the exact mechanisms guiding PRL/PAK1 signaling in breast cancer cells have not been fully elucidated. Our lab has previously demonstrated that PRL-activated tyrosine kinase JAK2 phosphorylates PAK1 on tyrosines 153, 201, and 285, and that tyrosyl phosphorylated PAK1 (pTyr-PAK1) augments migration and invasion of breast cancer cells.

**Results:**

Here we further investigate the mechanisms by which pTyr-PAK1 enhances breast cancer cell motility in response to PRL. We demonstrate a distinct reduction in PRL-induced FAK auto-phosphorylation in T47D and TMX2-28 breast cancer cells overexpressing wild-type PAK1 (PAK1 WT) when compared to cells overexpressing either GFP or phospho-tyrosine-deficient mutant PAK1 (PAK1 Y3F). Furthermore, pTyr-PAK1 phosphorylates MEK1 on Ser298 resulting in subsequent ERK1/2 activation. PRL-induced FAK auto-phosphorylation is rescued in PAK1 WT cells by inhibiting tyrosine phosphatases and tyrosine phosphatase inhibition abrogates cell motility and invasion in response to PRL. siRNA-mediated knockdown of the tyrosine phosphatase PTP-PEST rescues FAK auto-phosphorylation in PAK1 WT cells and reduces both cell motility and invasion. Finally, we provide evidence that PRL-induced pTyr-PAK1 stimulates tumor cell metastasis in vivo.

**Conclusion:**

These data provide insight into the mechanisms guiding PRL-mediated breast cancer cell motility and invasion and highlight a significant role for pTyr-PAK1 in breast cancer metastasis.

## Background

Prolactin (PRL) is a peptide hormone/cytokine that is typically secreted from the anterior pituitary gland, and has been found to be locally produced in various other organs such as the prostate, uterus, and mammary gland (for review [1]). Upon PRL binding, PRL-receptor (PRLR) dimerizes resulting in activation of the non-receptor tyrosine kinase JAK2 (Janus kinase 2) and subsequent downstream signaling cascades including signal tranducers and activators of transcription (STATs), mitogen activated protein kinases (MAPKs), including ERK1/2, and phosphoinositol-3 kinase pathways (for review [2]). PRL signaling at both an endocrine and paracrine/autocrine levels regulates a variety of physiological processes in an eclectic range of tissues (for review [3]). There is mounting evidence that PRL plays a significant role in breast cancer. The PRLR has been found in the vast majority of human breast cancers and PRL signaling has been implicated in breast cancer cell proliferation, survival, motility and angiogenesis (for review [2]). Furthermore, elevated circulating PRL levels have been positively correlated with breast cancer metastasis and PRLR-deficient mice have prevention of neoplasia progression into invasive carcinoma [4–7]. Importantly, PRL has been noted as a chemoattractant for breast cancer cells and augments tumor metastasis in nude mice [8, 9]. However, the exact mechanisms guiding PRL-induced cell migration and tumor metastasis are not fully understood.

We have implicated the serine/threonine kinase PAK1 (p21-activated kinase-1) as a substrate of PRL-activated JAK2 [10]. PAK1 has been associated with breast cancer progression (for review [11]). Aberrant expression/activation of PAK1 has been described in breast cancer as well as among several other cancers including brain, pancreas, colon, bladder, ovarian, hepatocellular, urinary tract, renal cell carcinoma, and thyroid cancers (for review [12]). The PAK1 gene lies within the 11q13 region and 11q13.5 → 11q14 amplifications involving the PAK1 locus are present in 17 % of breast cancers [13, 14]. PAK1 overexpression was observed in over half of observed breast tumor specimens [15] and PAK1 expression is correlated with tumor grade [16–18]. In transgenic mouse models, hyperactivation of PAK1 promotes mammary gland tumor formation [19]. Interestingly, overexpression of constitutively active PAK1 T423E in non-invasive breast cancer cells stimulates cell motility and anchorage independence [17], while expression of kinase dead PAK in highly invasive breast cancer cells significantly reduces cell invasiveness [20]. PAK1 kinase activity promotes directional cell motility and is a major regulator of the actin cytoskeleton (for review [11]). We have previously demonstrated that PRL-activated JAK2 directly phosphorylates PAK1 on tyrosines 153, 201, and 285 [10]. We have also demonstrated that tyrosyl phosphorylated PAK1 (pTyr-PAK1) enhances PRL-mediated cell invasion via MAPK activation and increased matrix metalloproteinase expression [21] as well as cell motility through increased phosphorylation of actin-crosslinking protein filamin A ([22]; reviewed in [23]). Additionally, PRL-induced pTyr-PAK1 is localized at small adhesion complexes at the cell periphery and regulates adhesion turnover in breast cancer cells, a process that is absolutely critical for cell motility [24].

Cell motility is essential in the regulation of many significant biological processes including embryogenesis, wound healing, and immune responses; however aberrant cell migration is present in malignant cancers and results in the establishment of tumors in distant tissues. Cell motility is a highly coordinated process that requires tight regulation of the actin cytoskeleton, cell-matrix adhesion turnover, and complex intracellular signaling cascades. The tyrosine kinase focal adhesion kinase (FAK) has been implicated as an important regulator of cell motility (for review [25]). FAK is localized to cell/matrix adhesions and is activated by integrin engagement to the extracellular matrix as well as by several other extracellular ligands (for review [26]). Auto-phosphorylation of FAK at tyrosine 397 (Y397) promotes FAK activation and recruits SH2- and SH3-domain containing proteins, most notably c-Src, leading to Src-mediated FAK activation and activation of Src/FAK signaling pathways, including the ERK MAPK signaling cascade (for review [26]). FAK activation has been most well implicated in the positive regulation of cell motility (for review [26, 27]). However, recently more evidence has demonstrated a controversial role for FAK as a negative regulator of cancer cell migration [28–30].

Here we extend our knowledge on the role for pTyr-PAK1 in PRL-induced breast cancer cell motility and invasion. We use T47D and TMX2-28 breast cancer cells stably overexpressing GFP, PAK1 WT, or tyrosyl phosphorylation-deficient mutant of PAK1 in which the three JAK2 phosphorylation sites have been mutated to phenylalanine (PAK1 Y3F). These cells were previously characterized in [22, 24] and [21]. We demonstrate here that tyrosyl phosphorylation of PAK1 in response to PRL regulates PTP-PEST-dependent FAK dephosphorylation, resulting in augmented breast cancer cell migration and invasion and proposed the mechanism explaining these findings. Furthermore, we provide in vivo evidence that PRL-induced pTyr-PAK1 increases breast cancer cell metastasis. Taken together, these data suggest that PRL-mediated pTyr-PAK1 is important in regulating the dynamic activation of FAK and subsequent breast cancer cell migration and invasion.

## Methods

### Antibodies and reagents

Polyclonal αpY397-FAK (Abcam), monoclonal αFAK (EMD Millipore), polyclonal αpS298-MEK (Cell Signaling), monoclonal αMEK (GeneTex), monoclonal αphospho-ERK1/2 (pT202/Y204) and polyclonal αERK1/2 (Cell Signaling), monoclonal αmyc (9E10, Santa Cruz Biotechnology), and αγ-tubulin (Sigma-Aldrich) were used for immunoblotting. Na_3_VO_4_ was purchased from Sigma. siRNA and primers for PTP-PEST were purchased from Santa Cruz Biotechnology. Control nontargeting siRNA was purchased from Cell Signaling. Human PRL was purchased from the National Hormone and Peptide Program (Dr. Parlow, National Institute of Diabetes and Digestive and Kidney Diseases).

### Cell culture

Prolactin receptor- and estrogen receptor-positive T47D cells stably overexpressing GFP, myc-tagged PAK1 WT, and myc-tagged PAK1 Y3F were described previously [22, 24]. T47D clones were maintained in RPMI 1640 medium (Corning Cellgro, Corning, Inc) supplemented with 10 % fetal bovine serum (FBS; Sigma-Aldrich) and insulin (Sigma-Aldrich). Prolactin receptor-positive but estrogen receptor-negative TMX2-28 cells (a variant of the MCF-7 breast cancer cell line [31]) and their clones stably overexpressing GFP, PAK1 WT or PAK1 Y3F were described previously [21] and maintained in DMEM supplemented with 10 % fetal bovine serum. The levels of overexpressed PAK1 WT and PAK1 Y3F were roughly estimated to be around 20-fold over the level of endogenous PAK1 in both T47D cells and TMX2-28 cells. MCF-7 cells were kindly donated by Dr. Ethier (University of Michigan) and T47D cells were purchased from the ATCC. TMX2-28 cells were kindly donated by Dr. Eisenmann (University of Toledo, OH).

### Assessing FAK, MEK, and ERK phosphorylation

T47D or TMX2-28 clones were seeded into 6-well dishes and deprived of serum for 72 h before treatment with or without PRL (200 ng/ml) for the indicated times. Cells were lysed and proteins were resolved by SDS-PAGE followed by immunoblotting with the indicated antibodies. Fold FAK, MEK, and ERK activation was assessed by densitometric analysis of αphospho-protein bands normalized to αtotal-protein bands using ImageJ software. To assess FAK activation in T47D clones in the absence of tyrosine phosphatase activity, cells were treated with 100 ng/ml of Na_3_VO_4_ for one hour before treatment with or without PRL (200 ng/ml) for the indicated times. Cells were lysed and proteins were resolved by SDS-PAGE followed by immunoblotting with the indicated antibodies. FAK activation was assessed by densitometric analysis of αpY397-FAK bands normalized to αFAK bands using ImageJ software.

### PTP-PEST knockdown

PTP-PEST siRNA or control nontargeting siRNA were transfected into T47D or TMX2-28 cells using Lipofectamine RNAiMAX (Invitrogen) according to the manufacturer’s instructions. The final concentration of the siRNA was 100 nM. Knockdown of PTP-PEST mRNA was assessed by RT-PCR method using PTP-PEST primers.

To assess PRL-induced FAK activation in the absence of PTP-PEST, T47D and TMX2-28 clones were transfected with PTP-PEST siRNA, deprived of serum for 48 h, and treated with or without PRL for the indicated times. Cells were lysed and proteins were resolved by SDS-PAGE followed by immunoblotting with the indicated antibodies.

### Cell viability

To assess cell viability in the presence of 100 ng/ml Na_3_VO_4_ for 48 h, equal numbers of T47D cells were resuspended in deprivation media (RPMI 1640 medium supplemented with 1 % BSA) with or without PRL (200 ng/ml) and Na_3_VO_4_ (100 ng/ml) then seeded into a 96-well plate. After 48 h, cells were subjected to the Vybrant® MTT Cell Proliferation Assay (Molecular Probes) according to the manufacturer’s instructions.

### Cell migration and cell invasion assays

Cell migration and cell invasion assays were performed as we described previously [21, 22]. Equal cell numbers of the T47D (1 × 10^6^ cells/chamber) or TMX2-28 (0.5 × 10^6^ cells/chamber) stable cell lines for each condition were placed in deprivation media with or without 100 ng/ml Na_3_VO_4_ in the upper chamber of a Boyden chamber (8.0 μm pores, Corning, Inc) (migration assay) or a Boyden chamber (8.0 μm pores), coated with Matrigel (BD Biosciences) (invasion assay). Deprivation media with or without 200 ng/ml PRL was placed in the lower chamber. Cells were allowed to migrate or invade for 48 h, after which the cells remaining in the upper chamber were removed from the upper chamber by a cotton swab. Cells from five separate fields that had migrated through the pores of the membrane to the underside of the filter were counted after fixation with 4 % formalin (Sigma) and staining with Differential Quik Stain (Polysciences, Inc). Brightfield images of migrated/invaded cells were acquired on an inverted Olympus IX81 microscope using LUCPlan FLN 40× objective lens and wide field WHN 10X eyepiece (Olympus, Tokyo, Japan).

To assess the effect of PTP-PEST knockdown on cell migration and invasion, T47D and TMX2-28 stable clones were transfected with PTP-PEST siRNA. After 24 h, cells were placed in deprivation media in the upper chamber of a Boyden chamber (migration assay) or a Boyden chamber coated with Matrigel (invasion assay). Cells were allowed to migrate/invade for 48 h and processed as described above.

### In vivo metastasis

TMX2-28 clones stably overexpressing GFP, myc-PAK1 WT or myc-PAK1 Y3F were inoculated directly into mammary fat pad of NSG (NOD/SCID/ IL2Rgamma) female mice. hPRL (20 μg/100 μl) was injected subcutaneously every other day for 8 weeks and mice were terminated in 12 weeks. 8 mice were used for TMX2-28 PAK1 clone, 6 mice for TMX2-28 PAK1 Y3F clone and 4 mice for TMX2-28 GFP clone. Mouse experimental procedures were performed in the animal research core of Lerner Research Institute, Cleveland Clinic (Dr. Lindner), and were approved by the Institutional Animal Care and Use Committee, Cleveland Clinic. The first half of tumors and lungs from mice was frozen and kept at −80 °C. Before use, the tissues were homogenized in RIPA buffer with protease inhibitors (50 mM Tris-HCl, 150 mM NaCl, 2 nM EGTA, 1 % Triton X-100, aprotinin 10 μg/ml, leupeptin, 10 μg/ml, pH7.5; 500 μL per 10 mg tissue) at 4 °C. Homogenized tissues were rotated in RIPA buffer for 1 h at 4 °C to ensure cell lysis. Samples were centrifuged at 10,000 g to pellet debris and protein concentration in supernatant was determined by Bradford assay. Proteins were separated by SDS-PAGE and transferred to PVDF membrane. Lysates of TMX2-28 PAK1 WT cells were loaded as a control for PAK1-myc position in the gels. Membranes were probed with anti-myc to detect myc-PAK1 WT or Y3F in the tissues and anti-tubulin for loading control. The second half of tumors and lungs was fixed with 10 % formalin and embedded in paraffin. Immunohistochemistry using paraffin-embedded sections was done as described previously [24]. Briefly, formalin-fixed, paraffin-embedded sections were boiled for 15 min in 0.01 M sodium citrate buffer (pH 6.0) to expose antigenic epitopes. Sections were blocked with 2.5 % normal horse serum for 30 min and then incubated overnight with anti-myc (1:100) or control pre-immune serum. The biotinylated secondary antibody was used followed by streptavidin horseradish peroxidase solution (R.T.U. Vectstatin universal quick kit, Vector Laboratories). The chromogen was 3,3’ diaminobenzidine (ImmPACT DAB kit, Vector Laboratories). Staining with pre-immune serum was negligible (not shown).

### Statistical analysis

Data from at least 3 separate experiments were pooled and analyzed using 1-way ANOVA plus Tukey’s honest significant difference test. Differences were considered to be statistically significant at *P* < 0.05. Results are expressed as the mean ± SE.

## Results

### Tyrosyl phosphorylated PAK1 negatively regulates FAK auto-phosphorylation

**Fig. 1:**
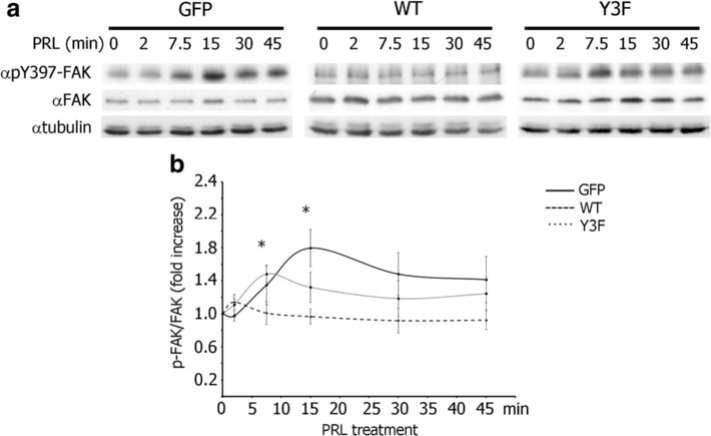
Tyrosyl phosphorylation of PAK1 negatively regulates PRL-induced FAK auto-phosphorylation. **a** Whole cell lysates (WCL) of T47D cells stably overexpressing GFP, PAK1 WT, or PAK1 Y3F treated with PRL (200 ng/ml) for the indicated times were probed for FAK auto-phosphorylation using αpY397-FAK antibody. The expression levels of γtubulin were used as an internal loading control. **b** Graph represents the densitometric analysis of the bands obtained for pY397-FAK normalized to total FAK for at least 3 independent experiments. The solid line represents T47D GFP cells, the dashed line represents T47D PAK1 WT cells, and the dotted line represents T47D PAK1 Y3F cells. Bars represent mean ± SE. *P < 0.05 compared with the same cells not treated with PRL

**Fig. 2:**
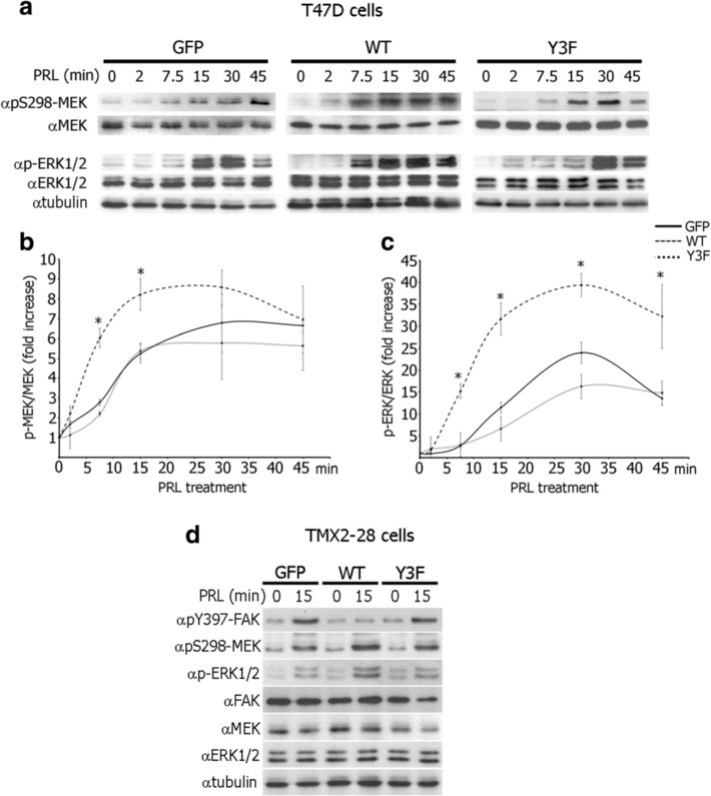
Tyrosyl phosphorylation of PAK1 promotes S298-MEK1 phosphorylation and ERK activation in response to PRL. **a** WCL of T47D cells stably overexpressing GFP, PAK1 WT, or PAK1 Y3F treated with PRL (200 ng/ml) for the indicated times were probed for MEK phosphorylation using αpS298-MEK and ERK1/2 activation using αphospho-ERK1/2 (pT202/Y204) antibodies. **b**, **c** Graphs represent the densitometric analysis of the bands obtained for phospho-MEK (**b**) or phospho-ERK1/2 (**c**) normalized to total MEK or ERK1/2, respectively, for at least 3 independent experiments. Bars represent mean ± SE . *P < 0.05 compared with cells expressing GFP with the same treatment. **d** WCL of TMX2-28 cells stably overexpressing GFP, PAK1 WT, or PAK1 Y3F treated with PRL (200 ng/ml) for the indicated times were probed with the indicated antibodies. The expression levels of γtubulin were used as an internal loading control

We have previously demonstrated that PRL promotes breast cancer cell motility in a pTyr-PAK1-dependent manner [22]. In an attempt to understand the pTyr-PAK1-dependent mechanism that regulates PRL-induced cell motility, we first examined the auto-phosphorylation of FAK in response to PRL, as FAK is an important regulator of cell motility (for review [25]). T47D GFP (control), PAK1 WT, or PAK1 Y3F (phospho-tyrosine-deficient mutant) clones were treated with PRL over a time-course and whole cell lysates (WCL) were analyzed for FAK auto-phosphorylation at Y397, which is critical for Src/FAK interaction and maximal FAK activation (reviewed in [32]). PRL treatment led to maximal FAK auto-phosphorylation in 15 min in control GFP cells (Fig. 1a, left blot; Fig. 1b, solid line). On the contrary, there was no significant Y397-FAK auto-phosphorylation in response to PRL in the PAK1 WT cells (Fig. 1a, middle blot; Fig. 1b, dashed line), suggesting that PRL-induced pTyr-PAK1 has a negative effect on FAK auto-phosphorylation. FAK was maximally auto-phosphorylated by PRL in 7.5 min in PAK1 Y3F cells (Fig. 1a, right blot; Fig. 1b, dotted line). Similar results were obtained in TMX2-28 (estrogen-receptor-negative sub-line of the MCF-7 breast cancer cells [31]) stably overexpressing GFP, myc-PAK1 WT, or myc-PAK1 Y3F (Fig. 2d, anti-pY397-FAK and anti-FAK blots) indicating that this finding was not restricted to T47D cells. It is important to note that PRL-dependent Y395-FAK phosphorylation was transient in both T47D GFP and T47D PAK1 Y3F clones. Our data suggest that tyrosyl phosphorylation of PAK1 in response to PRL promotes FAK dephosphorylation and tyrosines 153, 201 and 285 of PAK1 are responsible for this effect.

### Tyrosyl phosphorylation of PAK1 promotes S298-MEK1 phosphorylation and ERK activation in response to PRL

To uncover the mechanism by which pTyr-PAK1 may regulate FAK phosphorylation, we assessed S298-MEK phosphorylation and consequent ERK1/2 activation (dual phosphorylation of T202 and Y204 of ERK1/2 mediates ERK activity [33, 34]) in response to PRL because a PAK1/MEK/ERK signaling cascade has been implicated in Ras-mediated FAK dephosphorylation [30]. PRL promoted PAK1-dependent MEK phosphorylation 6-fold in as early as 7.5 min and maximal 8-fold MEK phosphorylation after 15 min in T47D PAK1 WT cells (Fig. 2a, middle blot, Fig. 2b). PRL also induced pS298-MEK signal in the T47D GFP and T47D PAK1 Y3F cells albeit slower and to a lesser extent when compared to the PAK1 WT cells (Fig. 2a, left and right blots, Fig. 2b). Subsequently, ERK1/2 was phosphorylated in response to PRL in all three T47D clones, however earlier and to a much greater extent in the PAK1 WT cells when compared to GFP and PAK1 Y3F cells (Fig. 2a and c). Similar results were obtained in TMX2-28 GFP, PAK1 WT and PAK1 Y3F cell clones (Fig. 2d, anti-pS298-MEK, anti-p-ERK1/2, anti-MEK and anti-ERK1/2 blots). These data suggest that PAK1 tyrosyl phosphorylation promotes PAK-dependent MEK phosphorylation and ERK activation in response to PRL.

### Protein tyrosine phosphatase inhibition rescues PRL-mediated auto-phosphorylation of FAK

**Fig. 3:**
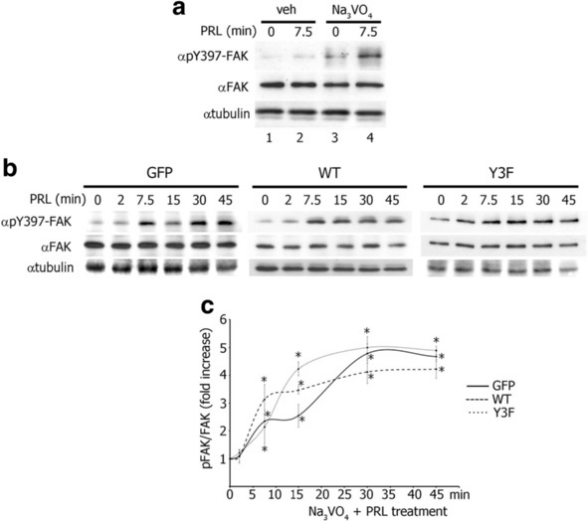
Protein tyrosine phosphatase inhibition rescues PRL-mediated FAK auto-phosphorylation in T47D WT cells. **a** Tyrosine phosphatase inhibition by Na_3_VO_4_ permits PRL-induced FAK auto-phosphorylation in PAK1 WT cells. WCL of T47D PAK1 WT cells treated with either vehicle (veh) or Na_3_VO_4_ (100 ng/ml) for 1 h before PRL (200 ng/ml) treatment were probed for FAK auto-phosphorylation by αpY397-FAK antibody. **b** FAK is auto-phosphorylation in T47D GFP, PAK1 WT, and PAK1 Y3F cells in response to PRL in the presence of Na_3_VO_4_. The cells were treated with Na_3_VO_4_ as in A and with PRL (200 ng/ml) for the indicated times. FAK auto-phosphorylation was assessed as in A. The expression levels of γtubulin were used as an internal loading control. **c** Graph represents the densitometric analysis of the bands obtained for pY397-FAK normalized to total FAK for at least 3 independent experiments. Bars represent mean ± SE. *P < 0.05 compared with the same cells not treated with PRL

In order to determine whether tyrosine phosphatases are involved in the negative effect of pTyr-PAK1 on FAK auto-phosphorylation, we assessed Y397- FAK phosphorylation in response to PRL in the presence or absence of Na_3_VO_4_, a tyrosine phosphatase inhibitor. T47D PAK1 WT cells were treated with vehicle or Na_3_VO_4_ for one hour before PRL treatment and WCL were assessed for pY397-FAK (Fig. 3). As expected, there was no PRL-mediated increase in FAK tyrosyl phosphorylation in vehicle treated cells (Fig. 3a, lanes 1 and 2). However, phosphatase inhibition led to a significant increase in both basal and PRL-induced FAK auto-phosphorylation (Fig. 3a, lanes 3 vs. 1 and 4 vs. 2). To confirm that phosphatase activity is important for the pTyr-PAK1-dependent effect of PRL on FAK dephosphorylation, all three T47D clones were subjected to a PRL time-course in the presence of Na_3_VO_4_ and WCL were assessed for Y397 FAK auto-phosphorylation (Fig. 3b, c). In the presence of Na_3_VO_4_, PRL treatment activated FAK in all three cell lines regardless of the status of PAK1 tyrosyl phosphorylation (Fig. 3b, c). Furthermore, FAK remained phosphorylated until the end of the PRL time-course in all three clones in the presence of Na_3_VO_4_.

**Fig. 4:**
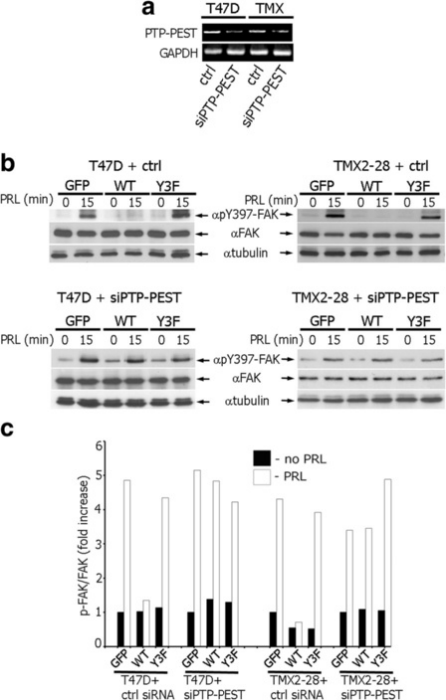
Silencing of tyrosine phosphatase PTP-PEST rescues FAK auto-phosphorylation in T47D and TMX2-28 cells. **a** PTP-PEST siRNA reduces PTP-PEST mRNA in T47D and TMX2-28 cells. T47D and TMX2-28 cells were transfected with either PTP-PEST siRNA or control non-coding siRNA (ctrl) and mRNA levels were assessed by RT-PCR using PTP-PEST-specific primers. GAPDH primers were used an internal control. **b** WCL of T47D and TMX2-28 clones transfected with control or PTP-PEST siRNAs and treated with PRL (200 ng/ml) for 0 or 15 min were probed for FAK auto-phosphorylation using αpY397-FAK antibody. The expression levels of γtubulin were used as an internal loading control. **c** Graph represents the densitometric analysis of the bands obtained for pY397-FAK normalized to total FAK

Next we aimed to determine whether tyrosine phosphatase PTP-PEST, which dephosphorylates FAK at Y397 [30], participates in PRL- and PAK1-dependent lack of FAK auto-phosphorylation. PTP-PEST silencing in T47D and TMX2-28 clones was confirmed by RT-PCR method (Fig. 4a). We performed siRNA-based silencing of PTP-PEST in T47D (Fig. 4b) and TMX2-28 (Fig. 4c) clones, treated the cells with or without PRL and assessed for Y397 FAK auto-phosphorylation. Indeed, PTP-PEST silencing rescued Y397-FAK phosphorylation in PAK1 WT cells to similar levels to that of GFP and PAK1 Y3F cells in response to PRL (Fig. 4b, c). On the contrary, there was no significant Y397-FAK auto-phosphorylation in response to PRL in the PAK1 WT clones transfected with control siRNA (Fig. 4b, c).

These data suggest that tyrosine phosphatase activity of PTP-PEST is responsible for the apparent lack of FAK auto-phosphorylation in response to PRL in PAK1 WT cells. Given the complexity of these signaling cascades, it is likely that additional signaling molecules are also involved in the modulation of FAK phosphorylation.

### Protein tyrosine phosphatase inhibition impedes PRL-mediated T47D and TMX2-28 cell migration and invasion

**Fig. 5:**
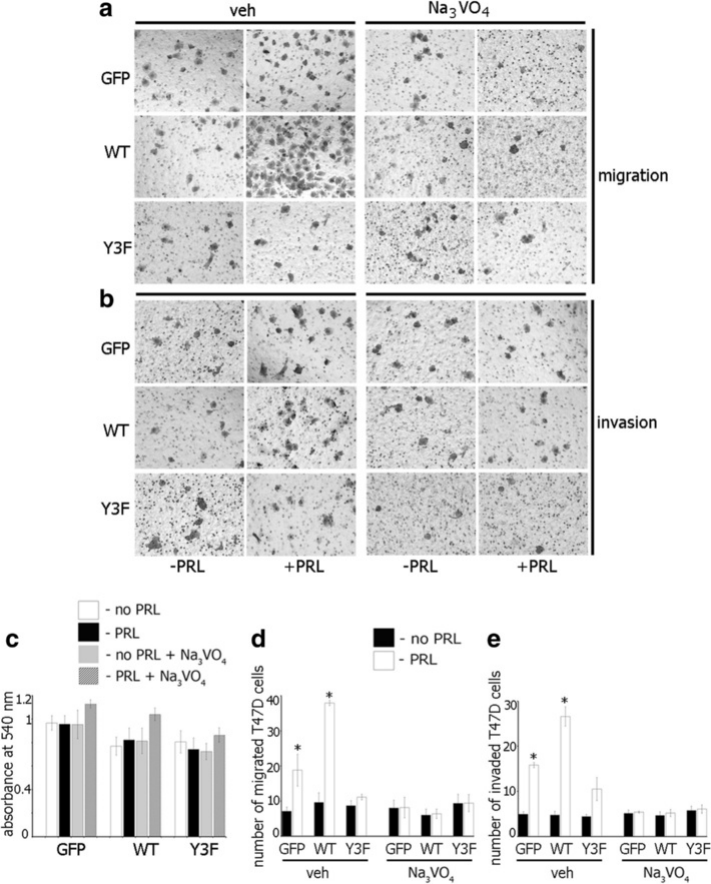
Protein tyrosine phosphatase inhibition impedes PRL-mediated T47D cell migration and invasion. **a**, **b** Equal amounts of T47D GFP, PAK WT, or PAK1 Y3F cells were loaded into the upper part of the Boyden chamber uncovered (**a**) or covered with Matrigel (**b**) with or without Na_3_VO_4_ (100 ng/ml). PRL (200 ng/ml) was added to the lower part. Representative brightfield images of the cells migrated/invaded to the lower chamber were taken in 48 h. A LUCPlan FLN 40X objective lens and wide field WHN 10X eyepiece on an inverted Olympus IX81 microscope were used. (**c**) Na_3_VO_4_ (100 ng/ml) treatment on T47D cells for 48 h has no cytotoxic effect. **d**, **e** The number of cells that migrated to the lower surface of the chamber toward PRL (white bar) or vehicle (black bar) after 48 h was counted and plotted. Bars represent mean ± SE. *P < 0.05 compared with the same cells not treated with PRL

To investigate whether tyrosine phosphatases regulate PRL/pTyr-PAK1-dependent T47D breast cancer cell migration, we examined migration of T47D clones in the presence and absence of PRL and Na_3_VO_4_ for 48 h. As dynamic tyrosyl phosphorylation events are crucial to many cellular processes, it was important to test whether phosphatase inhibition for an extended period of 48 h had any cytotoxic effect. The cell viability was assessed in serum deprived T47D cells treated with or without PRL and Na_3_VO_4_ (Fig. 5c). Na_3_VO_4_ had no significant cytotoxic effect on any of the three stable cell lines in the presence or absence of PRL (Fig. 5c). Next, the effect of tyrosine phosphatase inhibition on PRL-mediated cell migration was assessed using a transwell migration assay. Equal numbers of T47D GFP, PAK1 WT and PAK1 Y3F cells were seeded into the upper part of a Boyden chamber with or without Na_3_VO_4_ and PRL or vehicle were added to the bottom part. The number cells that migrated through the chamber towards PRL were counted (Figs. 5a and d). As we demonstrated previously [22], PRL stimulated cell migration to a greater extent in PAK1 WT cells when compared to GFP and PAK1 Y3F cells in the absence of Na_3_VO_4_ (Fig. 5d, veh). However, phosphatase inhibition by Na_3_VO_4_ completely abolished cell migration in response to PRL in all T47D clones (Fig. 5d, Na_3_VO_4_). These data suggest that phosphatase activity is required for pTyr-PAK1-induced cell migration.

Cell migration is a key step in cell invasion so we decided to assess the effect of phosphatase inhibition on cell invasion. Equal numbers of T47D GFP, PAK1 WT and PAK1 Y3F cells were seeded into the upper chamber of a Boyden chamber coated with Matrigel, in the presence of either Na_3_VO_4_ or vehicle. Deprivation media with or without PRL (200 ng/ml) was added to the lower chamber of the Boyden chamber. The number of cells that invaded through the Matrigel towards PRL was counted. As we demonstrated previously [21], PRL stimulated cell invasion to a greater extent in PAK1 WT cells when compared to GFP and PAK1 Y3F cells in the absence of Na_3_VO_4_ (Fig. 5b and e, veh). However, Na_3_VO_4_-mediated tyrosine phosphatase inhibition abolished cell invasion in response to PRL in all T47D clones (Fig. 5e, Na_3_VO_4_).

**Fig. 6:**
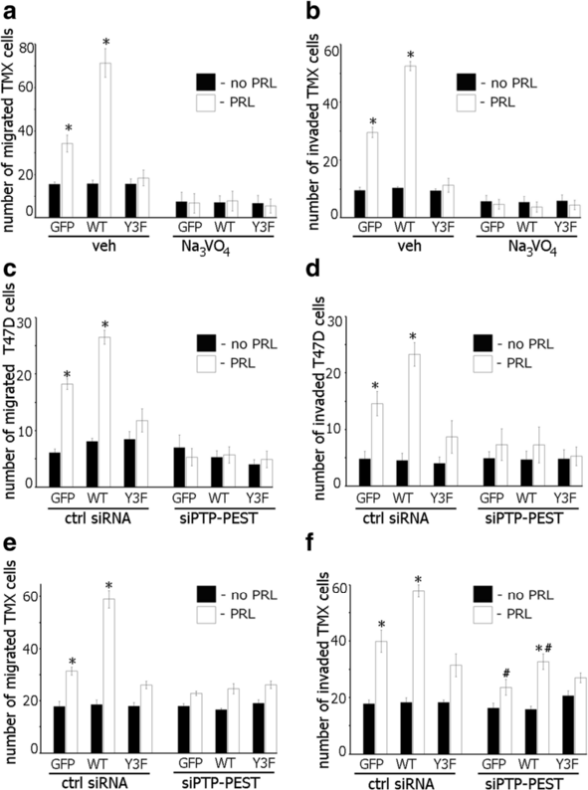
Silencing of the tyrosine phosphatase PTP-PEST reduces PRL-mediated cell migration and invasion. **a** Tyrosine phosphatase inhibition abolishes PRL-induced TMX2-28 cell migration **a** and invasion **b**. TMX2-28 GFP, PAK WT, or PAK1 Y3F cells were assessed as in Fig. 5. **c**–**f** Equal amount of T47D (**c**, **d**) or TMX2-28 (**e**, **f**) clones were transfected with either control or PTP-PEST siRNA and loaded into the upper part of the Boyden chamber covered (**d**, **f**) or not (**c**, **e**) with Matrigel. The number of cells that migrated/invaded to the lower chamber toward PRL (white bar) or vehicle (black bar) after 48 h was counted and plotted. Bars represent mean ± SE. *P < 0.05 compared with the same cells not treated with PRL. #*P* < 0.05 compared with the same cells treated with PRL but transfected with control siRNA (**f**)

To demonstrate that the role of PAK1 in PRL-mediated signaling is not limited to T47D cells, we assessed migration and invasion in the presence and absence of Na_3_VO_4_ in TMX2-28 clones. In TMX2-28 GFP and TMX2-28 WT cells PRL induced cell migration (Fig. 6a, veh) and invasion (Fig. 6b, veh) while in TMX2-28 Y3F cells did not (Fig. 6a and b, veh). However, Na_3_VO_4_ treatment abolished PRL-dependent cell migration and invasion in all TMX2-28 clones (Fig. 6a and b, Na_3_VO_4_)_._ Silencing of PTP-PEST also abolished PRL-dependent cell migration and invasion of all T47D clones (Fig. 6c and d) and migration of TMX2-28 clones (Fig. 6e). PRL-induced invasion of TMX2-28 control (GFP) and WT cells was significantly decreased by silencing of PTP-PEST although not completely abolished (Fig. 6f) suggesting that, in addition to PTP-PEST, other tyrosine phosphatases may participate in the pTyr-PAK1-dependent invasion of TMX2-28 cells.

### PAK1 tyrosyl phosphorylation stimulates PRL-induced tumor metastasis in vivo

**Fig. 7:**
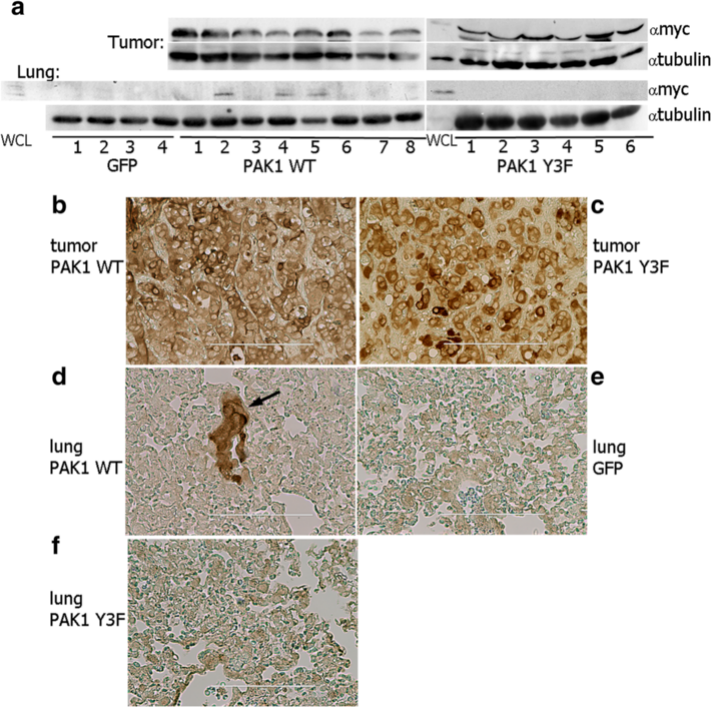
PAK1 tyrosyl phosphorylation stimulates PRL-induced tumor metastasis in vivo. **a** myc-PAK1 was detected in all tumor lysates isolated from myc-PAK1 WT and myc-PAK1 Y3F inoculated mice. PRL-induced tyrosyl phosphorylation of PAK1increased tumor metastasis, as 3 out of 8 lungs from WT mice contained myc-PAK1 (lanes 2, 4 and 5). No myc-PAK1 was detected in any of the lungs from the Y3F or GFP mice. Anti-tubulin antibody was used as a loading control. Whole cell lysate (WCL) of TMX2-28 PAK1 WT cells was loaded as a control for PAK1-myc position in the gels. **b**–**f** Representative images of myc-PAK1 detected with anti-myc in breast tumor (**b**, **c**) and lung tissue (**d**–**f**). myc-PAK1 was detected in breast tumors of myc-PAK1 WT- (**b**) and Y3F- (**c**) and lung of myc-PAK1 WT- inoculated mice (**d**) but not in lungs of GFP (**e**) or PAK1 Y3F-inoculated mice (**f**). The arrow highlights metastatic nodule. Counterstaining with hematoxylin was omitted. Scale bar is 200 μm in (**b**) and 100 μm in (**c**–**e**)

Cell migration is critical for tumor cell metastasis. In order to assess whether PRL-induced tyrosyl phosphorylation of PAK1 has a physiological effect on breast cancer metastasis, TMX2-28 stably overexpressing GFP, myc-PAK1 WT, or myc-PAK1 Y3F were inoculated in mouse mammary fat pads and mice were treated with PRL for 8 weeks. Tumors and lungs were harvested and homogenized and proteins were separated by SDS-PAGE and analyzed for myc-tagged PAK1 to indicate metastasis of the primary tumor into distant tissues. We focused on the primary tumor and the lungs, as the lungs are one of the most common sites for secondary tumor in patients with metastatic breast cancer. As expected, each primary tumor from all PAK1 WT and PAK1 Y3F mice was positive for myc-tagged PAK1 (Fig. 7a) while GFP cells do not produce tumors. Myc-tagged PAK1 was detected in 3 out of 8 lungs from the PAK1 WT mice while there was no detectable myc-PAK1 in any of the PAK1 Y3F or GFP mouse lungs (Fig. 7a). Tumors and lungs were also fixed and analyzed by immunocytochemistry (IHC) with anti-myc. Our IHC analysis revealed that anti-myc signal was detected in breast tumor (B) of myc-PAK1 WT- and myc-PAK1 Y3F-inoculated mice (C) as well as in lung of myc-PAK1 WT-inoculated mice (D) but not in lungs of control GFP- (E) or PAK1 Y3F-inoculated mice (F). These data provide first in vivo evidence that tyrosyl phosphorylation of PAK1 plays a significant role in PRL-induced breast cancer cell motility and metastasis, as only cells overexpressing PAK1 WT, but not phospho-tyrosine-deficient PAK1 Y3F, were able to migrate from the primary tumor to the lungs. Here we provide new insight into the mechanisms regulating PRL-dependent breast cancer cell metastasis.

## Discussion

The role of PAK1 in the regulation of cell motility is well documented (reviewed in [11]). The role of PAK1 in the regulation of cell adhesion is also well documented and at least one mechanism has been proposed ([35], reviewed in [36]). According to this mechanism, PAK1 phosphorylates paxillin on Ser273, leading to increased paxillin-GIT1 binding and adhesion turnover [35]. We have previously implicated PRL/JAK2-dependent tyrosyl phosphorylation of PAK1 in regulation of cell motility and invasion [21, 22]. We have also implicated pTyr-PAK1 in the regulation of breast cancer cell adhesion and demonstrated that phosphorylation of tyrsines 153, 201 and 285 of PAK1 regulates cell adhesion, contribute to maximal PAK1 kinase activity and increased ability to bind βPIX and GIT1 [24]. Here we extend our findings and demonstrate that pTyr-PAK1 phosphorylates MEK1 on Ser289 resulting in subsequent ERK1/2 activation. We also show that PRL-induced FAK auto-phosphorylation on Tyr397 is inhibited by pTyr-PAK1 and can be rescued by inhibiting tyrosine phosphatases and silencing tyrosine phosphatase PTP-PEST. These tyrosine phosphatase inhibitions abrogate cell motility and invasion in response to PRL. We hypothesize that pSer910-FAK recruits tyrosine phosphatase PTP-PEST to dephosphorylate pTyr397-FAK and thereby promotes cell motility as shown previously [30].

Dynamic of FAK phosphorylation is significant for cell motility. Previously, FAK activation has been demonstrated to positively regulate cell motility (for review [25–27]) however it is becoming evident that the role of FAK activation in cell migration is more complex. Silencing FAK using siRNA enhanced HeLa cell migration on collagen, and FAK dephosphorylation on Y397 by the tyrosine phosphatase PTP-PEST promoted Ras-induced cell migration in transformed NIH3T3-v-H-Ras cells [29, 30]. Cells with reduced FAK dephosphorylation had diminished cell motility [37] and overexpression of the tyrosine phosphatase LMR-PTP, which dephosphorylates FAK, enhanced cell motility [38]. Importantly, Zheng et al. implicated PAK1 in regulation of Ras-induced FAK dephosphorylation, as overexpression of constitutively active PAK1 T423E promoted FAK dephosphorylation while inhibition of PAK1 severely abolished FAK dephosphorylation at Y397 [30]. With agreement with these data, we have shown here that pTyr-PAK1 abolished PRL-dependent phosphorylation of Ser397-FAK.

We previously demonstrated that tyrosyl phosphorylation of PAK1 promotes both PAK1 kinase activity and protein-protein interaction capabilities (for review [23]). PAK1 directly binds to ERK in response to adhesion to fibronectin, and both PAK1 and ERK co-localize at nascent adhesions on the cell periphery [39]. Here, PAK1 can serve as a scaffold, bringing together Raf, MEK and ERK at cell/matrix adhesions and thereby stimulating ERK-dependent signal transduction [39]. In addition to PAK1 scaffolding activity, PAK1 promotes Raf activation by directly phosphorylated Raf on S338/339 [40, 41], and stimulates MEK/ERK binding and subsequent ERK activity by directly phosphorylating S298 on MEK1 [42–44]. Importantly, pS298-MEK has been shown to localize at peripheral adhesion complexes in response to cell adhesion to fibronectin [45]. Concurrently, we have demonstrated that tyrosyl phosphorylated PAK1 is localized at peripheral adhesion complexes in response to PRL and is responsible for proper adhesion turnover, an important process in cell migration [24]. This is important, as FAK is also localized at peripheral adhesion complexes and dynamic FAK localization and phosphorylation is important for proper adhesion turnover and cell migration (for review [25]). FAK localization to peripheral cell/matrix adhesions is dependent on its focal adhesion targeting (FAT) domain and binding to adhesion proteins paxillin and vinculin [46, 47]. Paxillin phosphorylation at Y31 and Y118 by FAK is necessary for cell migration and adhesion turnover [48–50], however, constitutive tyrosyl phosphorylation of paxillin impedes cell migration, and dephosphorylation of FAK by PTP-PEST is required for proper adhesion turnover in migrating cells [51]. Furthermore, overexpression of the dominant negative form of protein phosphatase LMR-PTP leads to FAK hyperphosphorylation and reduced cell motility [38] suggesting that complex regulation of FAK at adhesion complexes is necessary for proper cell migration. Phosphorylation of the FAK FAT domain on S910 and Y925 by ERK2 and Src, respectively, results in reduced FAK/paxillin binding and promotes adhesion turnover [52, 53]. Furthermore, pS298-MEK/ERK activation in NIH3T3 cells was shown to induce FAK dephosphorylation through ERK-mediated FAK S910 phosphorylation and resulting recruitment of tyrosine phosphatase PTP-PEST and thereby promote cell motility [30]. In this regard, PRL-induced tyrosyl phosphorylation of PAK1 and resulting adhesion localization could be creating localized PAK1/MAPK/FAK signaling at adhesion complexes and promoting adhesion turnover during cell migration.

In the present study we demonstrated that tyrosyl phosphorylation of PAK1 stimulates tumor cell metastasis in vivo. These data, combined with an animal study reporting prevention of neoplasia progression into invasive carcinoma in PRL receptor deficient mice [7], suggest that PRL is involved in the development of metastasis and tumor progression. Thus, our current data on pTyr-PAK1 regulation of FAK phosphorylation bring insight into the mechanism of PRL-stimulated motility of breast cancer cells.

## Conclusions

**Fig. 8:**
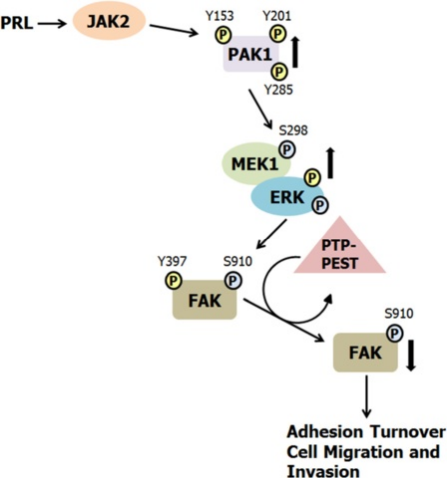
Proposed mechanism for the role of PRL-activated PAK1 in breast cancer cell migration. PRL binding to the PRLR results in activation of the non-receptor tyrosine kinase JAK2. JAK2 tyrosyl phosphorylates PAK1 on Y153, 201, and 285, enhancing PAK1 kinase and scaffolding activities. PRL treatment also leads to FAK auto-phosphorylation at Y397. Activated PAK1 phosphorylates MEK1 at S298, resulting in increased MEK1/ERK binding and enhanced ERK activity. Active ERK phosphorylates FAK at S910, leading to dephosphorylation of FAK at Y397 by the tyrosine phosphatase PTP-PEST as shown by Zheng et al. (2009). FAK dephosphorylation decreases FAK kinase activity and promotes adhesion turnover and breast cancer cell migration

Here we propose a mechanism by which PRL regulates motility of T47D and TMX2-28 cells through pTyr-PAK1, MEK/ERK and FAK that integrates our findings with previous studies (Fig. 8). In response to PRL, FAK is auto-phosphorylated and PAK1 is tyrosyl phosphorylated by JAK2, stimulating PAK1 kinase activity and increasing PAK1 protein-protein binding abilities. We show that tyrosyl phosphorylated PAK1 phosphorylates MEK at serine 298, resulting in MEK-mediated ERK1/2 activation. Activated ERK phosphorylates FAK at S910, leading to subsequent recruitment of PTP-PEST and dephosphorylation of Y397-FAK [30]. PRL-dependent down-regulation of FAK activity may promote focal contact turnover thereby promoting cell migration. Finally, we demonstrate for the first time that tyrosyl phosphorylation of PAK1 by PRL increases breast cancer cell metastasis in vivo.

## Abbreviations

ERK, extracellular signal-related kinase; FAK, focal adhesion kinase; JAK2, Janus kinase 2; MEK1, MAPK/ERK kinase 1; PAK1, p21-activated kinase 1; PRL, prolactin; WCL, whole cell lysate

## References

[1] Marano RJ, Ben-Jonathan N (2014). Minireview: extrapituitary prolactin: an update on the distribution, regulation, and functions. Mol Endocrinol.

[2] Clevenger CV, Furth PA, Hankinson SE, Schuler LA (2003). The role of prolactin in mammary carcinoma. Endocr Rev.

[3] Bernichtein S, Touraine P, Goffin V (2010). New concepts in prolactin biology. J Endocrinol.

[4] Holtkamp W, Nagel GA, Wander HE, Rauschecker HF, von Heyden D (1984). Hyperprolactinemia is an indicator of progressive disease and poor prognosis in advanced breast cancer. Int J Cancer.

[5] Bhatavdekar JM, Shah NG, Balar DB, Patel DD, Bhaduri A, Trivedi SN (1990). Plasma prolactin as an indicator of disease progression in advanced breast cancer. Cancer.

[6] Mujagic Z, Mujagic H (2004). Importance of serum prolactin determination in metastatic breast cancer patients. Croat Med J.

[7] Oakes SR, Robertson FG, Kench JG, Gardiner-Garden M, Wand MP, Green JE (2007). Loss of mammary epithelial prolactin receptor delays tumor formation by reducing cell proliferation in low-grade preinvasive lesions. Oncogene.

[8] Maus MV, Reilly SC, Clevenger CV (1999). Prolactin as a chemoattractant for human breast carcinoma. Endocrinology.

[9] Liby K, Neltner B, Mohamet L, Menchen L, Ben-Jonathan N (2003). Prolactin overexpression by MDA-MB-435 human breast cancer cells accelerates tumor growth. Breast Cancer Res Treat.

[10] Rider L, Shatrova A, Feener EP, Webb L, Diakonova M (2007). JAK2 tyrosine kinase phosphorylates PAK1 and regulates PAK1 activity and functions. J Biol Chem.

[11] Molli PR, Li DQ, Murray BW, Rayala SK, Kumar R (2009). PAK signaling in oncogenesis. Oncogene.

[12] Kumar R, Gururaj AE, Barnes CJ (2006). p21-activated kinases in cancer. Nat Rev Cancer.

[13] Bekri S, Adelaide J, Merscher S, Grosgeorge J, Caroli-Bosc F, Perucca-Lostanlen D (1997). Detailed map of a region commonly amplified at 11q13→q14 in human breast carcinoma. Cytogenet Cell Genet.

[14] Ong CC, Jubb AM, Haverty PM, Zhou W, Tran V, Truong T (2011). Targeting p21-activated kinase 1 (PAK1) to induce apoptosis of tumor cells. Proc Natl Acad Sci U S A.

[15] Balasenthil S, Sahin AA, Barnes CJ, Wang RA, Pestell RG, Vadlamudi RK (2004). p21-activated kinase-1 signaling mediates cyclin D1 expression in mammary epithelial and cancer cells. J Biol Chem.

[16] Salh B, Marotta A, Wagey R, Sayed M, Pelech S (2002). Dysregulation of phosphatidylinositol 3-kinase and downstream effectors in human breast cancer. Int J Cancer.

[17] Vadlamudi RK, Adam L, Wang RA, Mandal M, Nguyen D, Sahin A (2000). Regulatable expression of p21-activated kinase-1 promotes anchorage-independent growth and abnormal organization of mitotic spindles in human epithelial breast cancer cells. J Biol Chem.

[18] Holm C, Rayala S, Jirstrom K, Stal O, Kumar R, Landberg G (2006). Association between Pak1 expression and subcellular localization and tamoxifen resistance in breast cancer patients. J Natl Cancer Inst.

[19] Wang RA, Zhang H, Balasenthil S, Medina D, Kumar R (2006). PAK1 hyperactivation is sufficient for mammary gland tumor formation. Oncogene.

[20] Adam L, Vadlamudi R, Mandal M, Chernoff J, Kumar R (2000). Regulation of microfilament reorganization and invasiveness of breast cancer cells by kinase dead p21-activated kinase-1. J Biol Chem.

[21] Rider L, Oladimeji P, Diakonova M (2013). PAK1 regulates breast cancer cell invasion through secretion of matrix metalloproteinases in response to prolactin and three-dimensional collagen IV. Mol Endocrinol.

[22] Hammer A, Rider L, Oladimeji P, Cook L, Li Q, Mattingly RR (2013). Tyrosyl phosphorylated PAK1 regulates breast cancer cell motility in response to prolactin through filamin a. Mol Endocrinol.

[23] Hammer A, Diakonova M (2015). Tyrosyl phosphorylated serine-threonine kinase PAK1 is a novel regulator of prolactin-dependent breast cancer cell motility and invasion. Adv Exp Med Biol.

[24] Hammer A, Oladimeji P, De Las Casas LE, Diakonova M (2015). Phosphorylation of tyrosine 285 of PAK1 facilitates betaPIX/GIT1 binding and adhesion turnover. FASEB J.

[25] Mitra SK, Hanson DA, Schlaepfer DD (2005). Focal adhesion kinase: in command and control of cell motility. Nat Rev Mol Cell Biol.

[26] Parsons JT (2003). Focal adhesion kinase: the first 10 years. J Cell Sci.

[27] Hanks SK, Ryzhova L, Shin NY, Brabek J (2003). Focal adhesion kinase signaling activities and their implications in the control of cell survival and motility. Front Biosci.

[28] Schaller MD (2004). FAK and paxillin: regulators of N-cadherin adhesion and inhibitors of cell migration?. J Cell Biol.

[29] Yano H, Mazaki Y, Kurokawa K, Hanks SK, Matsuda M, Sabe H (2004). Roles played by a subset of integrin signaling molecules in cadherin-based cell-cell adhesion. J Cell Biol.

[30] Zheng Y, Xia Y, Hawke D, Halle M, Tremblay ML, Gao X (2009). FAK phosphorylation by ERK primes ras-induced tyrosine dephosphorylation of FAK mediated by PIN1 and PTP-PEST. Mol Cell.

[31] Fasco MJ, Amin A, Pentecost BT, Yang Y, Gierthy JF (2003). Phenotypic changes in MCF-7 cells during prolonged exposure to tamoxifen. Mol Cell Endocrinol.

[32] Schlaepfer DD, Mitra SK, Ilic D (2004). Control of motile and invasive cell phenotypes by focal adhesion kinase. Biochim Biophys Acta.

[33] Payne DM, Rossomando AJ, Martino P, Erickson AK, Her JH, Shabanowitz J (1991). Identification of the regulatory phosphorylation sites in pp 42/mitogen-activated protein kinase (MAP kinase). EMBO J.

[34] Zhang J, Zhang F, Ebert D, Cobb MH, Goldsmith EJ (1995). Activity of the MAP kinase ERK2 is controlled by a flexible surface loop. Structure.

[35] Nayal A, Webb DJ, Brown CM, Schaefer EM, Vicente-Manzanares M, Horwitz AR (2006). Paxillin phosphorylation at Ser273 localizes a GIT1-PIX-PAK complex and regulates adhesion and protrusion dynamics. J Cell Biol.

[36] Parrini MC (2012). Untangling the complexity of PAK1 dynamics: the future challenge. Cell Logist.

[37] Yu DH, Qu CK, Henegariu O, Lu X, Feng GS (1998). Protein-tyrosine phosphatase Shp-2 regulates cell spreading, migration, and focal adhesion. J Biol Chem.

[38] Rigacci S, Rovida E, Dello Sbarba P, Berti A (2002). Low Mr phosphotyrosine protein phosphatase associates and dephosphorylates p125 focal adhesion kinase, interfering with cell motility and spreading. J Biol Chem.

[39] Sundberg-Smith LJ, Doherty JT, Mack CP, Taylor JM (2005). Adhesion stimulates direct PAK1/ERK2 association and leads to ERK-dependent PAK1 Thr212 phosphorylation. J Biol Chem.

[40] Chaudhary A, King WG, Mattaliano MD, Frost JA, Diaz B, Morrison DK (2000). Phosphatidylinositol 3-kinase regulates Raf1 through Pak phosphorylation of serine 338. Curr Biol.

[41] Zang M, Hayne C, Luo Z (2002). Interaction between active Pak1 and Raf-1 is necessary for phosphorylation and activation of Raf-1. J Biol Chem.

[42] Frost JA, Steen H, Shapiro P, Lewis T, Ahn N, Shaw PE (1997). Cross-cascade activation of ERKs and ternary complex factors by Rho family proteins. Embo J.

[43] Coles LC, Shaw PE (2002). PAK1 primes MEK1 for phosphorylation by Raf-1 kinase during cross-cascade activation of the ERK pathway. Oncogene.

[44] Park ER, Eblen ST, Catling AD (2007). MEK1 activation by PAK: a novel mechanism. Cell Signal.

[45] Slack-Davis JK, Eblen ST, Zecevic M, Boerner SA, Tarcsafalvi A, Diaz HB (2003). PAK1 phosphorylation of MEK1 regulates fibronectin-stimulated MAPK activation. J Cell Biol.

[46] Tachibana K, Sato T, D’Avirro N, Morimoto C (1995). Direct association of pp125FAK with paxillin, the focal adhesion-targeting mechanism of pp125FAK. J Exp Med.

[47] Chen HC, Appeddu PA, Parsons JT, Hildebrand JD, Schaller MD, Guan JL (1995). Interaction of focal adhesion kinase with cytoskeletal protein talin. J Biol Chem.

[48] Petit V, Boyer B, Lentz D, Turner CE, Thiery JP, Valles AM (2000). Phosphorylation of tyrosine residues 31 and 118 on paxillin regulates cell migration through an association with CRK in NBT-II cells. J Cell Biol.

[49] Webb DJ, Donais K, Whitmore LA, Thomas SM, Turner CE, Parsons JT (2004). FAK-Src signalling through paxillin, ERK and MLCK regulates adhesion disassembly. Nat Cell Biol.

[50] Zaidel-Bar R, Milo R, Kam Z, Geiger B (2007). A paxillin tyrosine phosphorylation switch regulates the assembly and form of cell-matrix adhesions. J Cell Sci.

[51] Angers-Loustau A, Cote JF, Charest A, Dowbenko D, Spencer S, Lasky LA (1999). Protein tyrosine phosphatase-PEST regulates focal adhesion disassembly, migration, and cytokinesis in fibroblasts. J Cell Biol.

[52] Hunger-Glaser I, Fan RS, Perez-Salazar E, Rozengurt E (2004). PDGF and FGF induce focal adhesion kinase (FAK) phosphorylation at Ser-910: dissociation from Tyr-397 phosphorylation and requirement for ERK activation. J Cell Physiol.

[53] Katz BZ, Romer L, Miyamoto S, Volberg T, Matsumoto K, Cukierman E (2003). Targeting membrane-localized focal adhesion kinase to focal adhesions: roles of tyrosine phosphorylation and SRC family kinases. J Biol Chem.

